# Scientometric Overview of Coffee By-Products and Their Applications

**DOI:** 10.3390/molecules26247605

**Published:** 2021-12-15

**Authors:** Daniel D. Durán-Aranguren, Sebastian Robledo, Eduardo Gomez-Restrepo, Jorge W. Arboleda Valencia, Natalia A. Tarazona

**Affiliations:** 1 Product and Process Design Group, Department of Chemical and Food Engineering, Universidad de Los Andes, Carrera 1 No. 18A-10, Bogota 111711, Colombia; dd.duran10@uniandes.edu.co; 2 GESNE Research Group, Universidad Católica Luis Amigó, Tv. 51a # 67b 90, Medellin 050034, Colombia; sebastian.robledogi@amigo.edu.co; 3 Centro de Bioinformática y Biología Computacional de Colombia—BIOS, Cra. 15b # 61b, Manizales 170002, Colombia; 4 Core of Science, Calle 44 # 29b-28, Dosquebradas 661008, Colombia; 5 Doctorado en Biotecnología, Instituto de Biología, Facultad de Ciencias Exactas y Naturales, Universidad de Antioquia, Cl. 67 # 53-108, Medellin 050010, Colombia; eduardo.gomezr@udea.edu.co; 6 FITOBIOL Research Group, Instituto de Biología, Facultad de Ciencias Exactas y Naturales, Universidad de Antioquia, Cl. 67 # 53-108, Medellin 050010, Colombia; jwarboleda@gmail.com; 7 Centro de Investigaciones en Medio Ambiente y Desarrollo—CIMAD, Universidad de Manizales, Cra. 9a # 19-03, Manizales 170001, Colombia; 8 Institute of Active Polymers, Helmholtz-Zentrum Hereon, 14513 Teltow, Germany

**Keywords:** coffee by-products, applications, scientometric analysis, coffee pulp, coffee husk, coffee silverskin, spent coffee grounds

## Abstract

As coffee consumption is on the rise, and the global coffee production creates an excess of 23 million tons of waste per year, a revolutionary transition towards a circular economy via the transformation and valorization of the main by-products from its cultivation and preparation (Coffee Husk (CH), Coffee Pulp (CP), Coffee Silverskin (CS), and Spent Coffee Grounds (SCG)) is inspiring researchers around the world. The recent growth of scholarly publications in the field and the emerging applications of coffee by-products published in these scientific papers encourages a systematic review to identify the knowledge structure, research hotspots, and to discuss the challenges and future directions. This paper displays a comprehensive scientometric analysis based on 108 articles with a high level of influence in the field of coffee by-products and their applications. According to our analysis, the research in this field shows an explosive growth since 2017, clustered in five core applications: bioactive compounds, microbial transformation, environmental applications, biofuels from thermochemical processes, and construction materials.

## 1. Introduction

Coffee has become one of the most popular beverages [1], with a world coffee consumption of 166,346 thousand 60 Kg bags (~10 million tons) in the coffee year 2020/2021 (20 October to 21 September) [1]. World coffee exports amounted to 10.07 million bags in the same coffee year, from which 32.7% were supplied by Brazil, 19.3% by Vietnam, and 9.9% by Colombia, as the top three exporting countries. This industry has a current world trade of USD 30.9 B, while 59.8% was imported to the European Union (mainly Germany, which exported 28.3% of the total from the EU, Italy, and France), 28% to the United States of America, and 5.6% to Japan [2].

With such consumption rates worldwide, rising awareness about the environmental impact of residues from the coffee industry is evidenced by several scientific studies dedicated to the valorization routes of coffee by-products within the circular economy paradigm [3]. The fruit of coffee plants, referred to as coffee cherries, comprises the pericarp and the seeds, the latter harvested for human consumption. The pericarp includes the skin (exocarp), mucilage (mesocarp), parchment (endocarp), and the two seeds of the coffee cherry consisting of a peripheral spermoderm or “silverskin”, an endosperm (responsible for taste and aroma), and the embryo. Upon harvesting and processing, two main by-products are generated, coffee husk (CH) or coffee pulp (CP), which depends on the post-harvest method to transform the cherry into a green bean [4]. CH is obtained through the dry or semi-dry method (unwashed) and comprises all the pericarp layers, while CP is generated by the wet method (washed) and contains the mucilage and the skin of the cherry.

The green bean is then stored and roasted in place or transported to consumer countries for roasting, where the coffee silverskin (CS) is released from the bean as a by-product. The roasting process alters the bean’s chemical composition and determines the organoleptic properties and color of the roasted bean [5]. The preparation of coffee beverages consists of brewing ground roasted beans with hot water, which leaves a major by-product known as spent coffee grounds (SCG). Although one can already perceive the large proportion of residues generated in the coffee industry that might end up in landfills, the numbers are quite cruel. From a rough average of the published data, it is possible to estimate that one ton of coffee produced by the wet method generates ~0.5 tons of CP (wet weight), from which the pulp accounts for 40–50% of the fresh weight of coffee berries. In comparison, the dry method produces ~0.2 tons of CH (dried weight) from one ton of harvested coffee fruit. The roasting process generates one ton of silverskin for 120 tons of roasted beans (0.008 per 1 ton), and the brewing leaves between 0.5–0.7 tons of SCG per one ton of green bean (~0.35 tons per one ton of cherries) [6].

Over the last two decades, by-products from coffee consumption have grown as an independent field, receiving considerable worldwide attention from researchers [7,8]. Some researchers have described the main functional properties of coffee by-products. For example, Esquivel and Jiménez [8] explain the properties of each part of the cherry. Murthy and Madhava [9] show the process and standard value addition of coffee by-products. However, both studies are descriptive and subjective. Other studies focus on a specific part of the cherry bean. For instance, Narita and Inouye [10] provide an overview of CS chemical composition, biological activity, and its reuse; Campos-Vena et al. [11] review the SCG theory and propose future research, legislative frameworks, and policy recommendations [12]. Other studies were reported in the literature with more specific applications, such as in the food industry (tea, jam, juice, jelly, and others) [13]. Finally, recent studies show difficulties in transforming coffee by-products [14,15]. However, none of these papers use quantitative techniques to identify the topic’s importance, evolution, and intellectual structure.

These studies failed to provide a quantitative perspective of coffee by-products. In this vein, citation analysis is suggested to mitigate the limitations. For example, Kohn and Gordon [16] pointed out that citation analysis incorporates the importance and influence of papers throughout a ranking without the limitation to a set of specific journals or published years. Unlike other similarly growing fields, papers generated in this field have not yet been analyzed systematically to reveal the different coffee by-products. This gap is surprising because coffee is the most popular beverage in the world [17].

Although researchers have studied the by-products from coffee during the last two decades, only a few studies have tried to organize the academic literature to explain the development in the research of coffee by-products [13,14,15]. The insights of these works have tended to be fragmented and subjective compared with the results of objective, data-based scientometric approaches. Therefore, this research aims to apply a quantitative approach to identify the scientometric productivity and applications of coffee by-products.

## 2. Methodology

The paper is split into two-step methodological approaches to understand the research production and applications of coffee by-products in-depth. The first step presents a scientometric analysis to understand the scientific structure of the research field, and, in the second step, we analyze the trends in coffee by-product applications in detail.

### 2.1. Step 1: Scientometric Analysis

The term scientometric was coined by Nalimove and Mulchenko (1969) to describe the quantitative aspects of research production [18]. The scientometric analysis is useful for understanding the scientific production of a research topic, for example, the academic literature production through years, influential journals, and the most productive researchers. This leads to developing an objective overview of a research field because it is based on the quantitative analysis of a large number of academic papers. As an example, scientometric techniques were applied to map the academic social network of coffee certification research [19].

The search was conducted in 2020 in Web of Science (WoS) Core Collection (from 1980 to 2020) and Scopus (from 2001 to 2020). To identify the relevant papers, we split the search into four parts according to each by-product of the coffee production. For example, a general keyword search in the title was “byproduct* or by-product*” and “application* or utilizat* or commerc* or valorizat*”, then we added each by-product in the topic “coffee pulp”, “coffee husk”, “coffee silver skin” or “silverskin”, and “spent coffee grounds*”. All document formats were included such as articles, conference proceedings, books, and editorial material. Table 1 presents the papers found in each database with the specific by-product. Then, duplicate articles in WoS and Scopus were removed, resulting in a total of 177 items that matched the criteria and were included in this step. Merging Scopus and WoS is a novel approach and a recent tendency in scientometric papers [20], and it can be done easily with the Bibliometrix R package [21].

**Figure 1:**
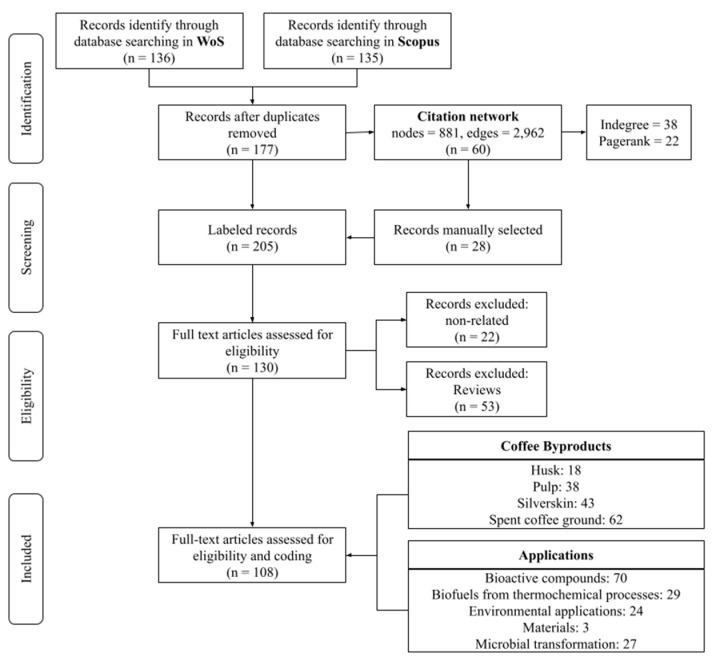
General flow diagram used to select papers based on the PRISMA guidelines.

Figure 1 shows the general process to select the most relevant academic literature of by-product coffee applications. We follow the PRISMA method to analyze only the most relevant literature [22]. In the next step, we created a citation network according to the process explained by Robledo [23] to identify relevant literature hidden in the references. The network has 881 nodes (articles) and 2962 edges (links), and we identified 60 relevant papers using indegree and page rank metrics [24]; then, we manually selected 28 papers related to our research topic. After removing duplicate papers, two authors screened the documents to identify reviews and non-related documents to remove them from the list. We found 22 papers not related to the main topic and 53 reviews. Reviews were removed from this step because they collect documents that create new knowledge, but reviews do not generate a new one. Finally, there is a dataset with 91,087 papers coded. The coded process is explained below. More details about the data analysis performed in this paper can be found in the supplementary materials.

### 2.2. Step 2: Analysis of Coffee By-Product Applications

In order to identify the main applications of each by-product, we created a table in which applications are divided into five categories: bioactive compounds, microbial transformation (and other biotechnological applications), environmental applications, biofuels from thermochemical processes, and materials. These categories were proposed by the two authors of this paper who are experts in this field, and they screened the papers to add the tags. Table 2 shows the categories and tags identified in the literature review with the results of this research. Results show that bioactive compounds are the most active applications, with 45% of the results. Specifically, food applications are the most studied in this category (49%). In contrast, biofuels from thermochemical processes are the second most dynamic category (31%), with solid fuels applications (42%) inside this group. This behavior shows the flexibility of coffee by-product applications.

## 3. Results

This section is split into two parts. The first one is an overview from a scientometric perspective analysis. We show the annual scientific production, authorship analysis, and journal analysis. The second one focuses on a temporal analysis of coffee by-product applications.

### 3.1. Scientometric Analysis

#### 3.1.1. Annual Scientific Production

**Figure 2:**
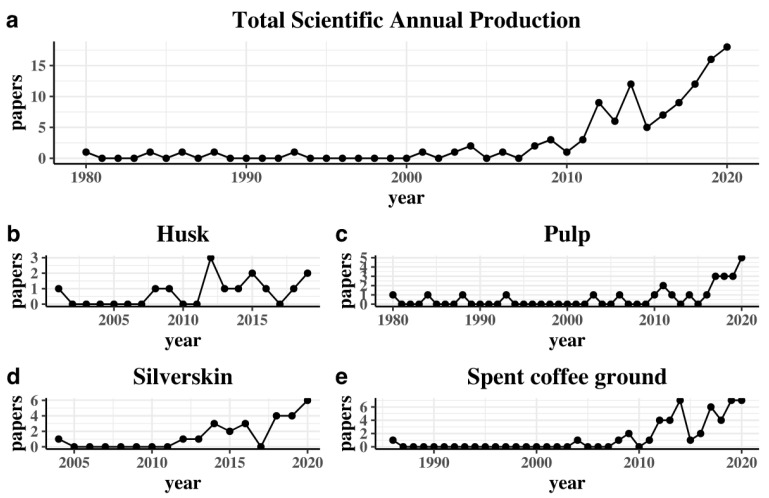
Scientific annual production of coffee by-products. (**a**) Total production, (**b**) Coffee Husk (CH), (**c**) Coffee Pulp (CP), (**d**) Coffee Silverskin (CS), (**e**) Spent Coffee Grounds (SCG).

Figure 2 shows the papers produced by year in each by-product. Figure 2a shows a steady tendency, and in the last five years scientific production increased sharply, and on average by 6.18% during the whole period. This behavior represents the current dynamic and the necessity to keep on researching. The first paper in this figure was published in 1980, and it is about how to transform pulp into juice [25]. Interestingly, the paper is almost 40 years old, but during its first 20 years the general scientific production was low (Figure 2c). All the figures show steady growth in the last three years due to the importance of transforming coffee residues.

Figure 2b reveals that the trend for the annual scientific production of husk by-product has fluctuated over the last ten years, with a hit in the bottom in 2013 but with an increase in 2020 with two papers. Silverskin applications show a more recent academic production starting in 2004 with the paper of Borrelli et al. [26], where they characterized the potential applications of coffee silverskin (see Figure 2d). Finally, the most productive academic applications are with SCG with 75 papers (39.9%) since 1986 with the work of Givens and Barber [27].

#### 3.1.2. Authorship Analysis

**Figure 3:**
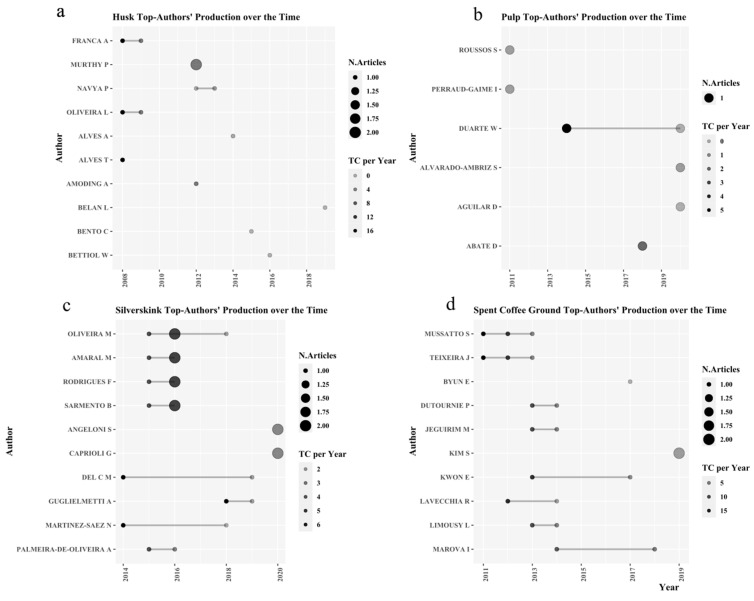
Author production per coffee by-product application over time for: (**a**) CH, (**b**) CP, (**c**) CS, (**d**) SCG.

In order to identify the most productive researchers in coffee by-product applications, we created Figure 3. This figure shows the most productive authors of each by-product through time with their average citations. Figure 1a shows the importance of professors Oliveira, Franca, and Alves in the husk applications. In 2008, they published a well-recognized paper that proposes an alternative use of husk as a bioadsorbent in the removal of cationic dyes [28]. This paper has 379 citations according to Google Scholar. In pulp applications, Dr. Duarte shows an application in bioethanol production [29], and this paper has been cited 84 times (Google Scholar). Figure 2c presents the more recent applications of silverskin and an interesting dynamic in their citations. Researcher Ana Palmeira-de-Oliveira has published four papers in the last seven years, and one of them has received particular attention for its possible application in cosmetics [30]. The authorship dynamic in SCG represents a diverse and prolific community. Professors Mussato and Teixeira have been working on several papers. For example, they identified the nutraceutical [31], food [32], and bioethanol applications of SCG [33] (see Figure 2d). These publications had received special recognition, with 845 citations in total according to Google Scholar.

**Figure 4:**
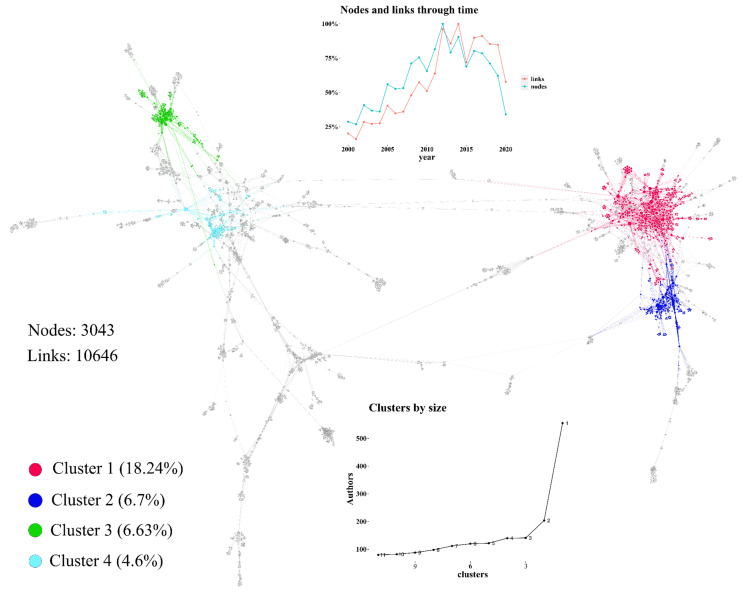
Academic social network of coffee by-product applications.

The importance of a research topic is a reflection of its academic social network (ASN). In this vein, we want to show the emerging social network of coffee by-products through time. We build this network by connecting authors when they published in the same paper. We included the same logic when using the references because most of the time, with only the first process, the ASNs generated are sparse and it is difficult to visualize the network structure [34]. Figure 4 shows a social academic network of 3042 researchers and 10,646 connections. We applied the Blondel et al. [35] algorithm to find the subgroups or clusters inside the ASN. The cluster-by-size inset figure shows the different sizes among the clusters with a starting tipping point in cluster 3 (the green one). Thus, cluster one (the red one) represents a mature ASN. Nodes and links through the time inset figure present an increasing number of new researchers through 2000 and 2010 because the blue line (nodes) is above the red one (links). Since 2010, there has been an increasing number of links that represent the consolidation of an ASN in coffee by-product applications.

#### 3.1.3. Journal Analysis

**Figure 5:**
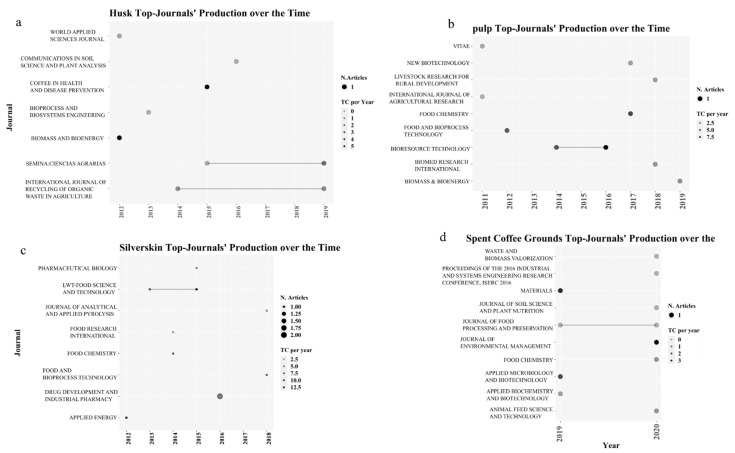
Journal production of each coffee by-product over time: (**a**) CH, (**b**) CP, (**c**) CS, (**d**) SCG.

This section explains the journal production through time (see Figure 5). All four coffee by-products present a rich and dynamic journal production. For example, Figure 5a shows two recent husk applications in the journals *Semina: Ciencias Agrarias* (Q3) and the *International Journal of Recycling of Organic Waste in Agriculture* (Q2). The first application is about feeding finishing lambs with husk [36] and the second is about composting [37]. Figure 5b shows the importance of the *Bioresource Technology* journal (Q1) in pulp applications. The first application is biodiesel [29] and the second is for the synthesis of lactic and organic acids [38]. The following graph (Figure 5c) shows that the *Drug Development and Industrial Pharmacy* (Q2) journal has two papers explaining the silverskin applications in bioactive compounds [30,39]. The last figure (Figure 5d) describes the dynamic in 2019 and 2020 SCG applications. In this graph, the *x*-axis only has two years due to the high number of journals that have been published in this by-product. The *Journal of Environmental Management* (Q1) presents an application of spent coffee grounds as biocatalysts and biochar components [40]. In general, the figures confirm the prolificacy and the quality of the recent publications in coffee by-product applications.

**Figure 6:**
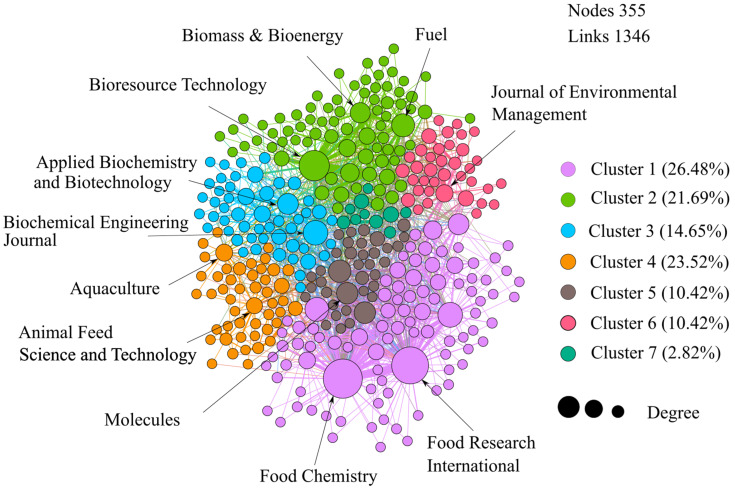
Journal citation network.

We generated a citation network of journals to identify the different clusters or groups of journals around a topic. Figure 6 shows an interesting result: most of the clusters identified in the citation network are related to the categories assigned. For example, bioactive compound applications are related to the journals of cluster 1, such as *Food Chemistry* and *Food Research International*. Microbial transformation applications, with journals like *Fuel*, *Biomass & Bioenergy*, and *Bioresource Technology*, are in cluster 2. Therefore, this result confirms how appropriate were the categories selected by the authors of this study.

### 3.2. Applications

It is evident that throughout the years the scientific community has expressed not only its concern about the disposition of the by-products generated during coffee processing, but also on how to extract value from this residual biomass. In this section, a temporal analysis of each one of the applications reported in the literature for coffee by-products is presented. Moreover, using the papers collected from the scientometric analysis, a more detailed description of the molecules that can be retrieved from coffee by-products and used in several applications is presented next.

#### 3.2.1. Bioactive Compounds

The research on the bioactive compounds from coffee by-products is mainly related to the use of their antioxidant, antimicrobial, and anticarcinogenic properties. For example, some phenolic compounds (like chlorogenic, ferulic acid, caffeic, and p-coumaric acids), alkaloids (like caffeine and trigonelline), and dipertenes (like cafestol and kahweol), and other secondary plant metabolites are present in coffee by-products [14,41]. However, it is important to mention that the presence and the abundance of these compounds would strongly depend on the geographical location in which the by-products are produced [42].

**Figure 7:**
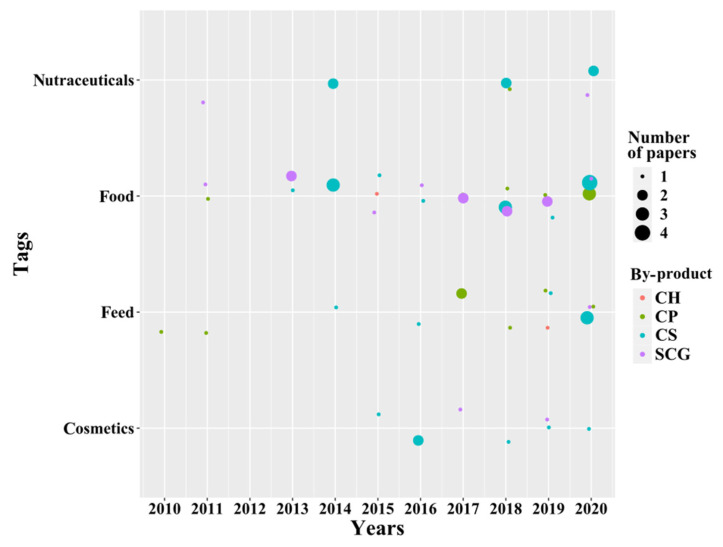
Temporal analysis of bioactive compound applications.

A temporal analysis of the bioactive compound’s applications of coffee by-products can be found in Figure 7. Four specific areas were identified: (i) nutraceuticals, (ii) foods, (iii) feeds, and (iv) cosmetics. In general, CS presents applications in all four tags (specific areas) with a more productive dynamic in foods. Moreover, the use of SCG in applications in the food industry throughout the time can also be highlighted. However, CH presents only two applications, one in foods (in 2015) and another in feeds (in 2019). Using this information, a more detailed discussion on the use of bioactive compounds from coffee by-products in each specific area is presented next.

##### Nutraceuticals

It is evident from Figure 7 that most of the research on nutraceutical applications has been done using CS. This by-product has been reported to contain bioactive compounds that have been used in dermocosmetics, since they have similar effects to hyaluronic acid and do not cause skin irritation [42]. Rodrigues et al. reported that CS extracts have a high antioxidant activity (related to its content of polyphenols), have antimicrobial properties against fungi and bacteria, and showed no cytotoxicity [43]. In another study, Rodrigues et al. formulated a hand cream containing 2.5% (*w*/*w*) of the extracts, which showed good stability and over time was able to preserve the properties of the bioactive compounds from CS [39]. What is more, Rodrigues et al. further explored the opportunities for the use of CS extracts by using them in a body cream formulation (topical cream) with similar benefits [30]. Moreover, it would be also possible to explore the use of CS extracts in other types of nutraceutical products since they have been reported to contain short chain fatty acids such as butanoic, propionic, and acetic acids that could help to decrease blood pressure and reduce fat absorption [44]. On the other hand, regarding the use of SCG, there is also a potential of the extracts of this by-product in nutraceuticals that could be further explored. Extracts from SCG with high contents of caffeine and chlorogenic acid were reported by Pettinato et. al, which could be interesting to use in cheaper and healthier products due to its biocompatibility with human cells [45]. Nonetheless, from the gathered information, it is possible to say that the application of CP and CH in nutraceuticals is yet to be explored.

##### Food

We identified 15 papers with applications of silverskin in food products. This could be attributed to its composition, since it has a high protein (~16–19%) and fiber content (~70–80%) [44]. Several studies have suggested the incorporation of CS into healthy foods due to its prebiotic, antioxidant, and antimicrobial properties [26,46,47,48]. Moreover, CS has over 40 odorants (volatile organic compounds), which could be used in food formulations, and it has a high content of polyphenols (such as 3-ethylphenol and the roasting-derived molecule 5-methyl-2-methoxyphenol), which could enhance the antioxidant properties of edible products [44]. CS has also been reported to contain significant amounts of feruloylquinic and caffeoylquinic acids, and around two times the caffeine content present in coffee beverages [15]. Furthermore, all of these bioactive compounds have been demonstrated to be bioaccessible and to have an enhancement in their antioxidant properties during digestion in the colon due to the action of the non-pathogenic bacteria [15]. What is more, due to the antimicrobial properties of CS bioactive compounds, the shelf-life time of these functional foods could be improved [15]. Due to its high fiber content [26], CS has been used as a source of dietary fiber in the formulations of baked goods such as bread [49,50] and cereal-based foods [51]. Moreover, due to the presence of chlorogenic acid and caffeine in CS, the possibility to formulate beverages that could help in weight control and fat reduction [52] that could diminish the risk of chronic metabolic diseases [52,53] has also been explored. These kinds of beverages using CS bioactive compounds have been formulated in combination with extracts from orange peel and cocoa bean shells [54].

The beneficial properties of including bioactive compounds into foods have also been reported using SCG [48,53,55]. A total of 12 papers have been published since 2011 related to the use of this by-product in edible goods (Figure 7). As happened with CS, due to the rich content of fiber (polysaccharides such as galactomannans and arabinogalactans) [56,57], amino acids [58], and polyphenols of SCG [31,58], this by-product has been incorporated into several bakery products such as biscuits [58] and cookies [59]. From SCG, the extraction of phytosterols (mainly β-sitosterol, stigmasterol, and campesterol), which are of great importance and have a significant global demand due to their use as food additives [60], was also reported. In a novel approach, the aroma compounds recovered from SCG were used to prepare an alcoholic beverage which retained most of the olfactory properties of this by-product [32].

A total of seven publications reporting the use of CP in foods were identified, most of them after the year 2018. This by-product has been fermented and used to produce distilled beverages due to its rich content of sugars and nutrients [61,62]. The alcoholic beverages (~38% *v/v* ethanol) contained several volatile compounds from CP, mainly ethyl esters such as ethyl dodecanoate and ethyl decanoate [61]. It has been used to produce CP beverages due to its content of sugars, polyphenols (rutin, gallic acid, protocatechuic acid, and chlorogenic acid), and caffeine [63]; for the same reasons, it has also been used in the formulation of infusions [64]. The approach proposed by Kusumocahyo et al., in which bioactive compounds from CP are obtained using spray drying, could be an interesting area for research, since this technology could preserve the antioxidant properties of the extract and allow its use in multiple products [65]. Moreover, the extracts from CP have been included in one study as an anti-inflammatory compound in a food supplement [66]. These extracts have been proven to have antifungal activities against Ascomycota and Mucoromycota fungi due to the presence of chlorogenic acid and caffeine, which makes CP extracts attractive for food preservation [67]. Regarding the application of CH in food products, only one paper was found which reported the cultivation of the fruiting bodies from the edible Basidiomycota fungi *P. ostreatus*. CH could serve as a substrate in which sodium selenite can be added and accumulated by the fungi, resulting in a food that could serve as a selenium supplement [68].

The information gathered from the scientometric analysis shows that the formulation of functional food products could be an interesting area for research that is already expanding, and presents itself as an alternative to valorize the biomass generated from the consumption of coffee beverages.

##### Feed

Related to feed applications, CP is highlighted as the main by-product used in animal nutrition. Moreau et al. proposed the use of fresh and ensiled CP to feed Nile tilapia due to the high protein and energy content that could be used by the fish; however, this study demonstrated that neither of those options were suitable to feed the fish and, on the contrary, that CP caused a reduction in feed utilization and compromised fish growth [69].This perplexing result could be attributed to the high content of bioactive compounds, especially polyphenols, which do not normally occur in the natural feed sources of the fish. In another example, dried CP was used to feed chickens, since it provides fiber, tannins, and caffeine to the animals. The study showed that this by-product can be included into chicken diets, but it would not provide nutrients since it has a low content of fat, protein, and ash [70]. Moreover, CP was used in mixtures with wheat straw to feed sheep, and it was demonstrated that this by-product could be used in replacement of concentrate feeds [71]. Other examples of the use of coffee by-products in animal feeds have been reported using SCG and CS. Díaz de Otálora et al. showed how the inclusion of SCG into concentrates enhanced the milk production of Latxa dairy ewes [72]. On the contrary, Givens et al. showed that the inclusion of SCG into ruminant feed was not a promising option [27]. Only one example reported the use of CH for animal feed, in which black soldier flies were reared and later used as fish feed [73]. However, the animals fed with these flies presented several health problems, which again could be possibly attributed to the content of polyphenols and caffeine in this by-product. From the gathered information, it is possible to deduce that the inclusion of coffee by-products into animal feed is strongly dependent on the species and their ability to digest the compounds present. More research is needed to expand the possibilities of using coffee by-products in feed.

##### Cosmetics

We found from the systematic process the use of CS and SCG in cosmetics. Earlier, the promising properties for cosmetic products of SCG [45] and the formulation of hand and body creams from CS extracts proposed by Rodrigues et al. [30,39] were described. Besides that, Kusumocahyo et al. proposed the formulation of a skin gel using the spray-dried extracts from CS, which further increased the antioxidant activity of the gel [74]. With that, it is important to note that the field of designing cosmetics from coffee by-products is yet to be explored and researched in detail.

#### 3.2.2. Microbial Transformations (and Other Biotechnological Applications)

**Figure 8:**
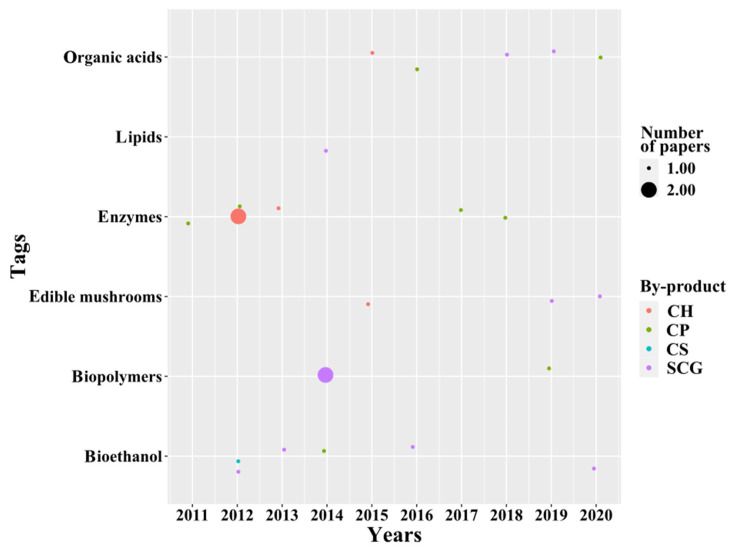
Temporal analysis of microbial transformation applications.

Microbial transformation for the valorization of coffee by-products is via the production of added-value building blocks, biomolecules, biomaterials, and bioenergy. One of the earliest and most popular applications in the category is focused on the use of by-products as a source of enzymes (Figure 8) with potential in biotechnology for the improvement of flavors and properties in food [75], and for extraction of phenolic acids (applications in Section 3.2.1, Bioactive Compounds) from different biomass streams using fungi and solid fermentation strategies [76]. Other popular applications in this field include the synthesis of organic acids, fermentation to produce bioethanol, the synthesis of biopolymers, and the use of coffee by-products to grow edible mushrooms (Figure 8). The temporal analysis presented in Figure 8 shows that SCG is the most used by-product for biotechnological applications, most likely because it is one of the most abundant and widely distributed by-products from the coffee industry. Moreover, it is followed by CH, which has been mainly used for enzyme production. A more detailed explanation of the recent findings found for the microbial transformation of coffee by-products is presented next.

##### Organic Acids

Fermentation of SCG and CP for the production of organic acids appears as a recent topic in this category. Promising strategies for the conversion of these by-products into lactic acid using yeast and lactic acid bacteria (LAB) have been presented [38,77,78]. The most common route to obtain lactic acid from SCG involves the acid hydrolysis of the by-product to depolymerize lignocellulose into reducing sugars (glucose, xylose, galactose, and arabinose), which are further used as a substrate for culturing LAB and other microbes with the capacity to produce lactic acid [38,77]. However, CP would not require the acid hydrolysis step since it already has a high content of fermentable sugars available. Lactic acid has been greatly used in the food, cosmetic, and chemical industries, and it is a valuable building block for the chemical synthesis of polylactic acid, a biodegradable polymer with promising mechanical properties for use in packaging applications. Nonetheless, the production of other organic acids (like acetic acid and succinic acid) using all four coffee by-products was not observed from the scientometric analysis, making it an interesting area to be explored in the future.

##### Lipids and Biopolymers

The content of free fatty acids in coffee oil extracted from SCG unlocked a new and relevant application for coffee by-products. Obruca et al. [79] showed that the oil extracted from SCG (~15 wt%) could be used for the microbial transformation of fatty acids into polyhydroxyalkanoates [79,80], which are biobased and biodegradable polymers (by depolymerases and lipases) spotlighted for the circular economy [81,82]. Interestingly, Achaby et al. [80] published in a recent paper the production of highly hydrated cellulose microfibrils from coffee pulp for applications in bioremediation as inexpensive adsorbent, e.g., the removal of pollutants from wastewater. New research in the field of biopolymers is expected given the urge to find new raw materials that could aid in the fight against synthetic and non-degradable plastics. It is important to note that the production of lipids using oleaginous yeasts grown in sugar-rich hydrolysates from coffee by-products was not evidenced from the retrieved papers, which makes it a novel area to investigate.

##### Enzymes

Coffee by-products have traditionally served both as a source of enzymes produced by the microorganisms growing on them and as substrate for enzymes with hydrolytic activity suitable for the depolymerization of the lignocellulosic compounds into fermentable sugars and phenolic compounds [75,76]. In total, eight papers were found in the first years of the scientometric analysis (see Figure 8). Nonetheless, there seems to be a decrease in the research efforts in this direction. Our analysis indicates that the use of enzymes is necessary to obtain several of the molecules reported in other categories (e.g., bioethanol, organic acids, and bioactive compounds), and for that reason should be explored further.

The use of enzymes is of utmost importance since they can release polyphenols and other bioactive compounds from coffee by-products without requiring the use of solvents and high temperatures. Promising results were shown when CP was incubated with a purified esterase from *Rhizoctonia solani*, releasing 100% of p-coumaric, 100% of caffeic, and 85% of ferulic acid from the biomass [76]. Similarly, feruloyl esterases from *Aspergillus niger* and *Aspergillus tamarii* have been used to hydrolyze the quinic esters from CP and release phenolic acids [83,84]. In addition, it is possible to obtain hydrolytic enzymes from coffee by-products. One example is the production of pectinases from CP, which has been done using microorganisms that naturally grow on this by-product [75], and also with the fungi *Aspergillus niger* [85]. The production of pectinase is relevant, since these enzymes are required in the food industry for juice clarification. On top of that, enzymes that are useful to hydrolyze structural carbohydrates have been produced. Murthy et al. evaluated the production of xylanase with *Penicillium* sp. (which hydrolyze the xylan backbone of hemicellulose) using all four coffee by-products [86]. In that study, it was found that CH resulted in the highest xylanase activity (~9500 U/g) [86]. In another study, endoglucanases from *Rhizopus stolonifer* were also produced from CH resulting in activities of ~390 U/mL [87]. Moreover, *Rhizopus stolonifer* was also used for cellulase production (~22,100 U/g) using CH and as substrates [88]. All of these hydrolytic enzymes are of great importance for the production of fermentable sugars that can be used in other fermentation processes. The information gathered allows us to infer that there is still room for research in the production and use of enzymes using coffee by-products as substrates. None of the collected articles from the scientometric analysis reported the use of SCG of CS for this purpose.

##### Edible Mushrooms

The academic literature captured on edible mushrooms was low; however, it is an application that has already been brought to the industry by several companies (GroCycle, Back to the Roots) sold under the name “organic mushrooms growth kit”. In the scientometric analysis, papers were found using SCG and CH for the production of fungal biomass from *Pleurotus ostreatus*, *Pleurotus eryngii*, and other mushrooms [89,90]. The use of coffee by-products has shown to have an impact on the yield and quality of the edible fungi, and in some cases to increase the content of beneficial minerals like selenium, as mentioned earlier [68].

##### Bioethanol

The sugar metabolism and the production of ethanol by yeast strains (e.g., *Saccharomyces cerevisiae*, *Pichia stipitis*, *Kluyveromyces fragilis*, and *Hanseniaspora uvarum*) has been exploited for the fermentation of sugar from hydrolysates produced by the acid hydrolysis of SCG, CP, and CH [29,33,55,91]. Some authors suggested a combined extraction of phenolic compounds and the production of ethanol, in which the extraction is carried out by either liquid-solid extraction, Soxhlet extraction, or microwave-assisted extraction. After the extraction, the remaining solids can be hydrolyzed to release C5 and C6 sugars, which are further fermented into bioethanol [55].The use of SCG and CS hydrolysates was thoroughly studied by Mussatto et al. in a study that compared the use of different yeast strains [33]. That study demonstrated that the highest ethanol productions and efficiencies were obtained when *S. cerevisiae* was cultivated in SCG hydrolysates. On the contrary, the use of CH hydrolysates was not satisfactory and would require the further concentration of the sugars [33]. Besides that, Burniol-Fignols et al. extracted phenolic compounds (mainly chlorogenic acid) from SCG and produced bioethanol (~3.9% *w*/*v*) from the hydrolysates [55]. In another example, the use of raw CH, which could contain reducing sugars to some extent, were used directly as a substrate for fermentation, resulting in good yields (0.29 g ethanol/g CH) [91]. Moreover, another study reported the use of raw CP (rich in fermentable sugars) to produce bioethanol, also with promising yields (0.41g ethanol/g CP) using the yeast *Hanseniaspora uvarum* [29]. Other authors have highlighted the valorization opportunities when the sequential coproduction of biodiesel and bioethanol is performed from the lipids and sugars extracted from SCG [92]. One interesting approach was reported by Battista et al., who proposed the use of SCG under a biorefinery concept in which the solid fraction obtained after the extraction of coffee oil and the production of ethanol was used to produce biogas [93]. This biorefinery also produced bio-oil, biodiesel, and PHA. With all of that, it is evident that the study of bioethanol production from coffee by-products has been an area of continuous interest throughout the years, and on which researchers could still produce more value in the future.

#### 3.2.3. Environmental Applications

**Figure 9:**
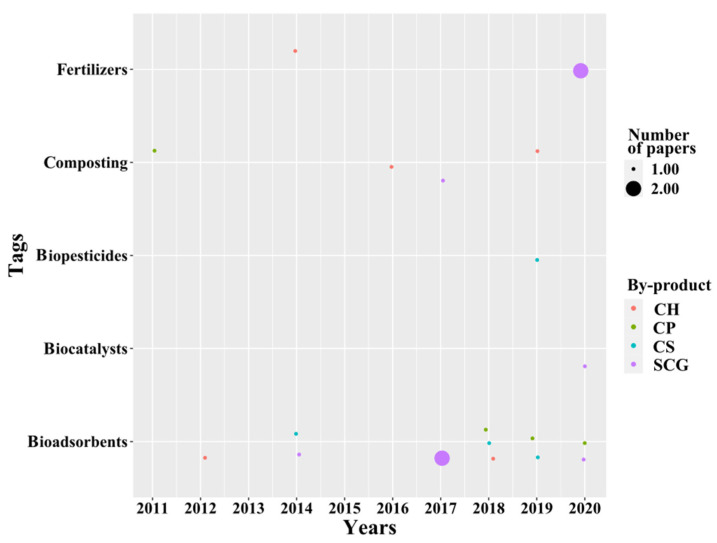
Temporal analysis of microbial transformation applications.

As can be seen in Figure 9, environmental applications of coffee by-product show the major use of all the by-products as bioadsorbents for, e.g., the removal of contaminants from wastewater [94]. Other applications include their use as a composting enhancer, as biofertilizers, and as pesticides. Each one of these applications is discussed in the following paragraphs.

##### Fertilizers

According to the literature, fertilizing with CH and SCG is a good cropping practice to contribute to the sustainability of agriculture [95,96]. In the work of Zoca et al., CP, CH, and composted CH were evaluated as fertilizers, since they contain the important mineral potassium (K) [97]. This study showed that CH composted for 40 weeks increased the release of K and could be used in soils as an amendment. Besides that, biochar from CH have been demonstrated to be the most productive amendments (due to their high soluble ash content) in contrast to the biochar of other residues, such as maize cobs, eucalyptus wood, and rice husks [98]. The use of CH and their biochar as soil amendments could reduce fertilization costs and prevent environmental degradation by recycling the nutrients that would be lost by leaching or runoff from the stored residue for prolonged times [97]. Moreover, SCG have been used to store stone pine seedlings, and it was found that mixtures of 10–20% SCG and sand increased the germination time and enhanced the characteristics of the seedlings by supplementing K, N, Mg, and P [95]. Similarly, water extracts from SCG and tea leaves have been used as the primer agents (growth inducers) of pepper [96]. It appears that coffee by-products have a rich composition of inorganic compounds that could further expand the possibilities for their utilization. More studies are needed to evaluate how they could be included as valuable raw materials for the formulation of green fertilizers. 

##### Composting

Due to the rich composition of coffee by-products in terms of carbohydrates, bioactive compounds, and proteins, they suitable for composting. Applications in this area were found for CH, CP, and SCG. In a study, CP and CH were successfully composted alone or co-composted with other degradable fractions of municipal solid waste in different ratios, producing very mature compost and contributing to the availability of iron and potassium [37]. Substitution of commercial growing media with, e.g., 10% CP showed a significant increase in the aerial biomass, seedling height, and the number of nodes per tomato plant [99]. CH was reported as being part of a mixture of a compost that enhanced the biomass growth of eucalyptus, which included chicken manure, sewage sludge, pine bark, coconut fiber, and sawdust [100]. Finally, SCG have also been co-composted together with olive mill waste and phosphogypsum, resulting in an increment of ~55% of potato yield [101]. 

##### Biopesticides

We found only one paper in biopesticides applications with CS. Thligene et al. [102] demonstrated that silverskin significantly reduced the nematode population in tomato harvests. As mentioned in the section on bioactive compounds, the presence of several polyphenols, terpenes, and caffeine in CS (that are not usually present in the feed sources of these nematodes) could be the reason for why the extracts act as pesticides and, what is more, how they could also provide protection to the fruit due to their antimicrobial properties [47].

##### Bioadsorbents and Biocatalysts

This is a rich area in coffee by-product applications, with a total of 13 papers. The raw coffee by-products and their activated carbons have been prepared for this purpose. The use of SCG, CH, and CS has been studied in the removal of methylene blue (MB) [28,103,104], usually evaluated as a model contaminant. Franca et al. used untreated SCG and CH to remove MB and verified their efficacy as adsorbent of this cationic dye [103]. The maximum value of the uptake capacity obtained for SCG adsorbing MB was comparable to values encountered in the literature for other untreated agricultural by-products and wastes [103]. In another study, SCG and CS were modified with magnetite nanoparticles showing high adsorption capacity of MB, with CS being better than SCG for that purpose [104]. Moreover, activated carbon from SCG and other coffee by-products has been used to remove MB and phenol, since it has an enhanced surface area that increases adsorption [105,106]. These activated carbons have demonstrated their usefulness to remove challenging pollutants such as As(V) and Mn(II) [94,107].

Furthermore, only one paper was retrieved in the category of biocatalysts, which used the biochar obtained from the pyrolysis of SCG to catalyze the degradation of sulfamethoxazole (SMX) by persulfate activation [40]. The obtained biochar (pyrolyzed for h at 850 °C) was reported to have a surface area of 492 m^2^/g, making it ideal to provide the surface area necessary to catalyze the degradation reaction [40]. It is important to highlight that the biochar adsorbed and provided the surface area for the degradation of SMX, a widely used antibiotic that can be found as a pollutant in several water sources [40]. Since this is a novel application, it would be interesting to study in more detail how different biochars from coffee by-products could be used in these kinds of catalysis processes.

#### 3.2.4. Biofuels from Thermochemical Processes

**Figure 10:**
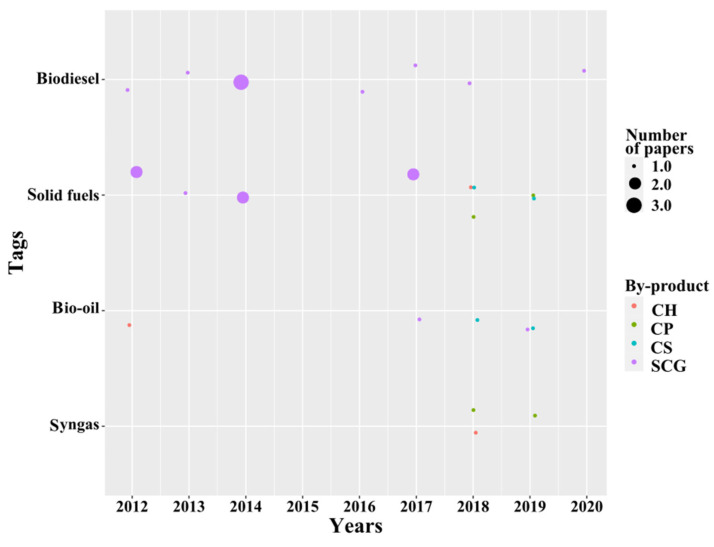
Temporal analysis of biofuels from thermochemical processes.

According to Figure 10, the temporal analysis shows that there is a homogeneous development of biofuels from thermochemical processes with recent findings. In the figure, it is possible to observe that most research has focused on the production of biodiesel and solid fuels from SCG. Nonetheless, since 2018 there has been an increase in the study of other coffee by-products (CH, CP, and CS) in applications more related to pyrolysis and gasification. In the following paragraphs, the reader will find a detailed explanation of the specific areas of research that were identified.

##### Biodiesel

Due to the high oil content in SCG, biodiesel has been studied as an attractive alternative for its valorization. SCG oil can represent from 10% up to 30% of the dry weight of the biomass [108,109,110,111], which would depend highly on the brewing technique, the variety of the feedstock, and the extraction method [111]. Moreover, it has been suggested that biodiesel from SCG oil has better stability due the abundance of antioxidant compounds in its composition. SCG oil is composed mostly of linoleic acid (37.3%), palmitic acid (35.8%), with small amounts of oleic acid (13.9%), stearic acid (8.1%), arachidic acid (3.2%), and others [108,109,110,111,112]. Other authors have reported a similar composition of SCG oil with polyunsaturated fatty acids representing more than 50% of the total fatty acids [109,110,111,112,113]. 

Haile et al. found that using a mixture of hexane and isopropanol (50:50% vol) resulted in the highest yields of oil in comparison with hexane and ether alone [108]. In general, the oil extracted from SCG by Haile et al. had an HHV of 38.22 MJ/kg, which indicates that it can be used for direct combustion; however, this alternative would be problematic, since ash deposits could be formed due to the high viscosity of the oil (0.917 g/cm^3^ at 15 °C) [108]. In terms of its suitability for biodiesel production, SCG oil has a high cloud point (11 °C) and requires acid pretreatment before its transformation into biodiesel to esterify the oil due to its acidity and high saponification value (167.28 mg KOH/g). However, it has a high flash point (>200 °C) and low rancidity, making SCG ideal for safety and storage. 

Biodiesel from SCG oil was obtained by Haile et al. using first an acid pretreatment (HCl 10% *w*/*w* of fatty acids, methanol to fatty acids molar ratio 20:1, 600 RPM, 54 °C, 90 min) and then a base-catalyzed transesterification process (methanol to oil ratio 9:1, 600 RPM, 54 °C, 90 min, 1%*w*/*w* KOH) [108]. A yield of 82% biodiesel was achieved with 6.8% of the total lost during the acid pretreatment. This biodiesel is within the standard limits (ASTM), except for their acid and carbon residue values due to the high content of unsaturated fatty acids in the extracted oil [108,113]. Similar yields between 80% and 90% have been reported by other authors [5]. Biodiesel is usually purified with hot water at 55°C to remove the residual catalyst, glycerol, methanol, and soap. Sulfuric acid is added in a second wash for further neutralization, and later anhydrous sodium sulfate is used to dry this biofuel.

##### Solid Fuels

A solid biofuel that is a product from pyrolysis is biochar. Biochar from CS has a LHV of ~21 kJ/kg, which is considerably higher than CS alone (~16.7 kJ/kg) [114]. Additionally, SCG have been reported to result in biochar with an HHV of ~31MJ/kg [110]. In both cases, the potential of this biofuel for energy production through incineration could be used and should be studied further since it could provide the energy for the drying of coffee beans. There are few reports focused on this potential application. 

SCG has an HHV of 22–24 MJ/kg, which suggests its potential for pelletization [105,110,113]. Pelletization is a process in which dried biomass is agglomerated by applying pressure to form a solid pellet that is usually combined with a binder to improve the stability. Jeguirim et al. compared the production of pellets from SCG and a mixture of SCG (50%) and sawdust (50%), and found that even though SCG and the mixture had similar thermal properties (LHV between 17.6 and 18.2 kJ/kg), the blend resulted in better pellets since its composition resulted in an earlier combustion [115]. A useful alternative is to use the residues that result from biodiesel production from SCG to produce pellets with the solid waste after the oil and the remnant glycerin retrieved from the biodiesel washing that serves as a binder [108]. SCG pellets are an attractive biofuel since they can be sold for their use in domestic and industrial (small-scale) heating.

##### Bio-Oil

Bio-oil is produced by pyrolysis, a thermochemical process in which the biomass is transformed in a low oxygen atmosphere at temperatures between 300 °C and 700 °C [116]. Yields of bio-oil are highly dependent on the process conditions and the composition of the biomass used. For example, using CS at temperatures around 280 °C (10 min residence time) resulted in a bio-oil with a low pH (due to the presence of acetic acid) and rich in phenolics, amino compounds (peptides, pyridines, and pyrroles), and caffeine dissolved in reaction water (a product of the pyrolysis reaction). These hydrophilic compounds result mainly from the degradation of structural carbohydrates. Low-temperature conditions result in an incomplete degradation, and given that CS has a low content of lignin, the amount of non-aqueous compounds produced was low and without an evident phase separation [114]. In another work, CS pyrolyzed at 560 °C and an N_2_ flow rate of 49 mL/min yielded 15.2% of the organic phase (non-aqueous) [106]. The bio-oil obtained was rich in phenols (which can be used as biofuel additives) and nitrogen compounds, and contained hydrocarbons (saturated, non-saturated, and aromatic) which could be used in the production of second-generation biofuels. These compounds can be retrieved by using liquid–liquid extraction (LLE), as Santos Polidoro et al. performed by using dichloromethane as a mass separating agent [106]. SCG have been used to produce bio-oil through pyrolysis. This by-product was evaluated by Lazzari et al. at 650°C, 10 min, and 100 mL/min of N_2_, in a system that required condensation at -10°C with a mixture of water and ethylene glycol, which allowed the retrieval of a bio-oil rich in phenols, nitrogen compounds, ketones, and aldehydes (e.g., furfural with potential as liquid biofuel) [117]. Yields of bio-oil from SCG have been reported to be between 50% and 60% [111].

##### Syngas

Syngas can be produced through a thermochemical process called gasification, in which biomass can be oxidized at high temperatures (800–1200 °C) to produce a mixture of hydrogen, carbon dioxide, carbon monoxide, and low amounts of hydrocarbons (methane, ethane, and others) that can be used in energy generation [81]. This process leaves some amounts of biochar and tar as by-products. One example is the work presented by Orosco et al., in which coffee pulp and CS syngas production was compared using modified fixed-bed gasifiers originally designed for wood chips [118]. The syngas obtained from coffee pulp and CS can be used to replace liquified petroleum gas (LPG), which is commonly used during the drying process of coffee beans. Orosco et al. demonstrated that this replacement can be done and that the fuel gas can maintain the required drying temperature (~50 °C) [118]. Torres et al. evaluated coffee pulp gasification and proposed a model to represent the process. The syngas obtained from coffee pulp was composed (in mole fraction) by hydrogen (15–20%), carbon dioxide (15–20%), methane (10–20%), carbon monoxide (20–25%), and nitrogen (20–30%) [119].

#### 3.2.5. Materials and Others

##### Construction Materials

**Figure 11:**
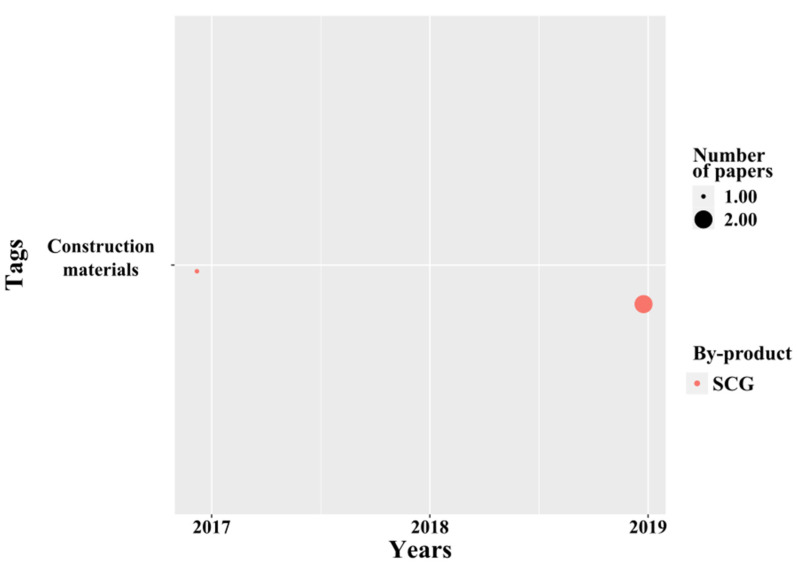
Temporal analysis of materials.

We identified few but recent papers about the material applications of coffee by-products (Figure 11). Currently, the materials that are extracted for construction are obtained from both renewable and non-renewable sources. More recently, the use of recycled materials in civil engineering has gained attention. The optimal mix of several coffee by-products demonstrated that SCG, CS, and RH ash could be used as sustainable materials in pavement applications [120]. Later, Andreola et al. experimented in clay ceramic aggregates with outstanding results, while SCG were tested as a pore-forming agent that partially replaces red clay in the material formulation before firing. The substitution of red clay, under this innovative application, showed that the light aggregates obtained have properties for possible uses in urban areas, such as green roofs as a drainage layer, as well as agricultural ones. In addition, it is aligned with the Italian standard on soil amendment. Results are promising for the elaboration of high-quality lightweight clay aggregates for drainage purposes [121]. Moreover, Yoo et al. [89] supported these results with thermal performance experiments. The storage and discharge of thermal energy are among the most efficient and reliable approaches to reducing energy consumption. Energy efficiency is one of the main problems of climate change globally. For this, phase change materials (PCM) are an answer to this need using the by-products of coffee, as they can provide a high energy storage density. The PCM application can be used in buildings by saving energy and reducing temperature fluctuations [122].

## 4. Conclusions

This paper identifies the main applications of coffee by-products through a literature review using scientometric techniques. We split the results into two sections. The first one presents the scientometric analysis. This part identified the growing production in the topic and the academic community involved around it. SCG have the richest academic production in coffee by-product applications; however, CS and CP have had a higher rate of production in recent years. Author analyses identified the community around the topic. This community has been growing and consolidating through time. Furthermore, the journal analysis showed the strong groups of papers around the well-defined topics.

The second section presents a detailed temporal analysis of the coffee by-product applications in bioactive compounds, microbial transformations, environmental, biofuels from thermochemical processes, and materials. In bioactive compounds, food is the most dynamic application using all the coffee by-products. However, regarding microbial transformation, the academic production is different, and it is split into diverse applications. Environmental applications are a relatively new topic in coffee by-products with an interesting production in bioabsorbents, but few in pesticides and biocatalysts. Biofuels from thermochemical processes present a solid production in which the use of SCG for biodiesel stands out throughout the analyzed years. Finally, we identified only three papers about material applications in the construction field, and all of them using SCG. More research must be performed to better understand how different valorization alternatives can be coupled to maximize the number of products obtained sequentially from coffee by-products, perhaps under the biorefinery concept.

## Figures and Tables

**Table 1:** Keyword search process.

Keyword Search	By-Product	WoS	Scopus	Results
Title = (byproduct* or by-product*) AND Title = (application* or utilizat* or commerc* or valorizat*)	Topic = (coffee pulp)	26	33	36
	Topic = (coffee husk)	16	21	25
	Topic = (coffee “silver skin”) OR (silverskin)	30	34	35
	Topic = (spent coffee grounds)	64	47	79
Total		136	125	177

**Table 2:** Categories and tags proposed to identify by-products applications.

Categories	Tags	Total Tags	Total Categories
Bioactive Compounds	Cosmetics	8 (11%)	72 (45%)
	Feed	18 (25%)	
	Food	35 (49%)	
	Nutraceuticals	11 (15%)	
Microbial Transformation	Bioethanol	7 (24%)	29 (18%)
	Biogas	1 (3%)	
	Biopolymers	3 (10%)	
	Edible mushrooms	3 (10%)	
	Enzymes	9 (31%)	
	Lipids	1 (3%)	
	Organic acids	5 (17%)	
Environmental Applications	Bioadsorbents	15 (58%)	26 (16%)
	Biocatalysts	1 (4%)	
	Biopesticides	1 (4%)	
	Fertilizers	6 (23%)	
Biofuels from Thermochemical Processes	Bio-oil	3 (12%)	31 (19%)
	Biodiesel	5 (16%)	
	Solid fuels	10 (32%)	
	Syngas	13 (42%)	
Materials	Construction	3 (10%)	3 (2%)

## Data Availability

Data of the review is available from the authors and the data analysis is available online at https://github.com/srobledog/coffee_by_products (accessed on 10 December 2021).

## Supplementary Materials

The data analysis is available online at https://github.com/srobledog/coffee_by_products accessed on 10 December 2021.

## Sample Availability

Data of the review is available from the authors.
